# Differential human gut microbiome assemblages during soil-transmitted helminth infections in Indonesia and Liberia

**DOI:** 10.1186/s40168-018-0416-5

**Published:** 2018-02-28

**Authors:** Bruce A. Rosa, Taniawati Supali, Lincoln Gankpala, Yenny Djuardi, Erliyani Sartono, Yanjiao Zhou, Kerstin Fischer, John Martin, Rahul Tyagi, Fatorma K. Bolay, Peter U. Fischer, Maria Yazdanbakhsh, Makedonka Mitreva

**Affiliations:** 1 McDonnell Genome Institute, Washington University, St. Louis, MO 63108 USA; 2 Department of Parasitology, Faculty of Medicine, Universitas Indonesia, Jakarta, Indonesia; 3 Public Health and Medical Research, National Public Health Institute of Liberia, Charlesville, Liberia; 4 Department of Parasitology, Leiden University Medical Center, Leiden, The Netherlands; 5 Microbial Genomics, The Jackson Laboratory for Genomic Medicine, Farmington, CT USA; 6 Department of Medicine, Washington University School of Medicine, St. Louis, MO USA

**Keywords:** Helminth, Nematode, Microbiota, Metagenome, Parasite, Intestine, 16S rRNA gene

## Abstract

**Background:**

The human intestine and its microbiota is the most common infection site for soil-transmitted helminths (STHs), which affect the well-being of ~ 1.5 billion people worldwide. The complex cross-kingdom interactions are not well understood.

**Results:**

A cross-sectional analysis identified conserved microbial signatures positively or negatively associated with STH infections across Liberia and Indonesia, and longitudinal samples analysis from a double-blind randomized trial showed that the gut microbiota responds to deworming but does not transition closer to the uninfected state. The microbiomes of individuals able to self-clear the infection had more alike microbiome assemblages compared to individuals who remained infected. One bacterial taxon (*Lachnospiracae*) was negatively associated with infection in both countries, and 12 bacterial taxa were significantly associated with STH infection in both countries, including *Olsenella* (associated with reduced gut inflammation), which also significantly reduced in abundance following clearance of infection. Microbial community gene abundances were also affected by deworming. Functional categories identified as associated with STH infection included arachidonic acid metabolism; arachidonic acid is the precursor for pro-inflammatory leukotrienes that threaten helminth survival, and our findings suggest that some modulation of arachidonic acid activity in the STH-infected gut may occur through the increase of arachidonic acid metabolizing bacteria.

**Conclusions:**

For the first time, we identify specific members of the gut microbiome that discriminate between moderately/heavily STH-infected and non-infected states across very diverse geographical regions using two different statistical methods. We also identify microbiome-encoded biological functions associated with the STH infections, which are associated potentially with STH survival strategies, and changes in the host environment. These results provide a novel insight of the cross-kingdom interactions in the human gut ecosystem by unlocking the microbiome assemblages at taxonomic, genetic, and functional levels so that advances towards key mechanistic studies can be made.

**Electronic supplementary material:**

The online version of this article (10.1186/s40168-018-0416-5) contains supplementary material, which is available to authorized users.

## Background

The gut microbiota of healthy older children and adults is complex but relatively stable in composition and function [1]. The gut microbiota has a large impact on human physiology, and has been demonstrated to modulate immune function, growth [2–4], metabolism, and overall health [5]. Helminths that reside in the gut can directly affect the immune system [6–8] or can indirectly influence the immune system through their effects on the gut microbiota and on the intestinal mucosa. Children are often exposed to helminth infections prior to the stabilization of their microbiota (up to 42% by 3 years of age [9]), increasing the risk of a lifetime disruption of normal microbial community development that can lead to long-term dysbiosis (possibly due to a reduced ability to extract nutrients from food for host absorption) [10]. Understanding the detailed relationship between helminths and the microbiota will provide important insights into how to reduce the negative impacts of helminth infection through simple nutritional strategies to supplement anthelminthic treatment as an integrated therapeutic tool [10]. However, little is known about the interactions between bacteria, the most prevalent soil-transmitted helminths (STHs; hookworms (*Necator americanus* and *Ancylostoma duodenale*], large roundworm [*Ascaris lumbricoides*], and whipworm [*Trichuris trichiura*]), and the human gut.

The massive number of infected individuals (1.5 billion), constant reinfections, highly variable drug efficacies, and disappointing treatment success rates for some species (e.g., whipworms [11]) make STHs one of the most important causes of chronic morbidity in the world. Despite massive efforts to control STHs [12], their prevalence remains high because of high rates of reinfection (i.e., 94, 82, and 57% for *Ascaris*, whipworm and hookworm, respectively; [13]). Furthermore, drug resistance (frequently against all three classes of anthelmintics) is common in veterinary helminthes [14], and resistance to montepantel (the latest anthelmintic introduced to the market) arose within 4 years of its release [15]). These problems of drug resistance may eventually pose a risk in humans [16]. This is especially important since according to WHO recommendations, the current target is to reduce moderate and heavy infections to less than 1% among school children [17], requiring high treatment coverage and increasing the selection for drug resistance in human nematode populations. Therefore, developing an integrated and sustainable STH management strategy for humans is urgently needed. Given recent progress in microbiome-driven therapeutics [18], gut bacterial genomics could offer insights that have eluded decades of mechanism-based and classic pharmacologic approaches to treatment, and may present opportunities for enhancing deworming efforts and understanding helminth-microbiome interactions.

The role of commensal bacteria in STH infections is not well understood, but it is recognized that active cross-kingdom talk exists (mainly based on animal studies, e.g., [19–25]), However, studies in humans have been very limited to date, with only two published cross-sectional studies [26, 27]. In addition, results from these studies were inconsistent. The study from Ecuador did not identify any association between STHs and the fecal microbiome [27] while the study from Malaysia found significantly higher microbiota richness in feces from humans with whipworm infections [26]. More studies are needed to determine if this discrepancy is related to confounding factors, such as the different geographical regions, different helminth species, the depth of sequencing, or the platform used (2 k 454/Roche vs. 19 k Illumina reads per sample). Overall, it has been noted that strong conclusions about the nature of helminth interactions with the gut microbiome have been limited by the small number of studies, variations in sampling and analysis techniques, and small sample sizes within each study [28].

Here, we greatly expand on the existing knowledge of interactions between the human gut microbiome and STHs by precisely defining the microbial ecology underlying the STH infections in two very distinct geographic regions (Liberia and Indonesia) and identifying conserved STH-associated taxa despite the very different gut microbiome structure among individuals from these regions. To determine the association among the STHs and the gut microbiome, we considered only moderately/heavily infected and uninfected individuals (with precisely-quantified infection status). We successfully identified bacterial taxa positively and negatively associated with single and/or multiple worm infections. Furthermore, we compared the longitudinal changes in microbiomes from a double-blind randomized trial, identifying bacterial taxa that respond to deworming, while taking the time and treatment effects into consideration. Reconstruction of the metabolic pathways of the microbial communities on a subset of the longitudinal samples revealed functions differentially affected by deworming. The results provide novel (and much more comprehensive) understanding of the cross-kingdom interactions and provide a solid foundation for designing experiments to determine mechanistic insight of these interactions during infections and after deworming.

## Results

**Fig. 1:**
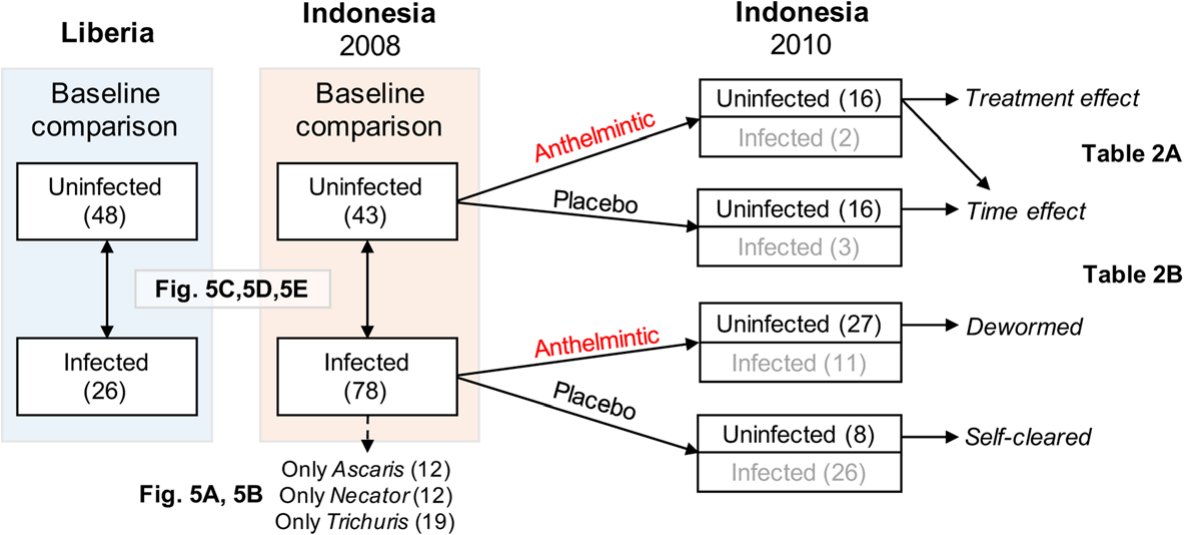
Sample set comparisons. Samples are excluded if they only have a low-level STH infection. Figures corresponding to each analysis are indicated

Our sample set included 402 fecal samples from 250 individuals from different villages in Liberia (*n* = 98; Table 1, Additional file 1: Table S1) and Indonesia (*n* = 304; Table 1, Additional file 2: Table S2). All samples were sequenced using targeted metagenomics (V1 V3 hypervariable region of 16S rRNA gene), and relevant sample set comparisons discussed below are shown in Fig. 1. A subset of samples (*n* = 24; Additional file 3: Table S3) were sequenced using a metagenomic shotgun approach. The STH species endemic in the studied areas included *Ascaris lumbricoides* (“*Ascaris*”), *Necator americanus* (“*Necator*”), and *Trichuris trichiura* (“*Trichuris*”). Other intestinal helminths such as *Ancylostoma duodenale*, *Enterobius vermicularis*, or *Strongyloides stercoralis* were not detected in the study population.

**Table 1:** Characteristics of 16S samples from Liberia and Indonesia

Category		Country of origin		
		Liberia	Indonesia	
			2008	2010
Total Number of samples		98	152	152
Number of villages sampled		8	3	3
Sex of individuals	Male	45	68	84
	Female	53	68	84
Age of individuals	Average	26	27	29
	Median	17.5	28	30
	Min. (90th percentile)	6	6	8
	Max (90th percentile)	59	49	51
Nematode presence* according to qPCR (< 28CT)	*Ascaris*	18	23	18
	*Necator*	8	32	15
	Trichuris**	1	42	34
	Any Infection	26	78	50
	Multiple infections	4	16	14
	Zero infection	48	43	83

*Moderate to heavy presence

**Trichuris identified by formol-ether concentration in Indonesia

### Diagnostic accuracy and precise worm burden quantitation drive accurate microbiome characterization

**Fig. 2:**
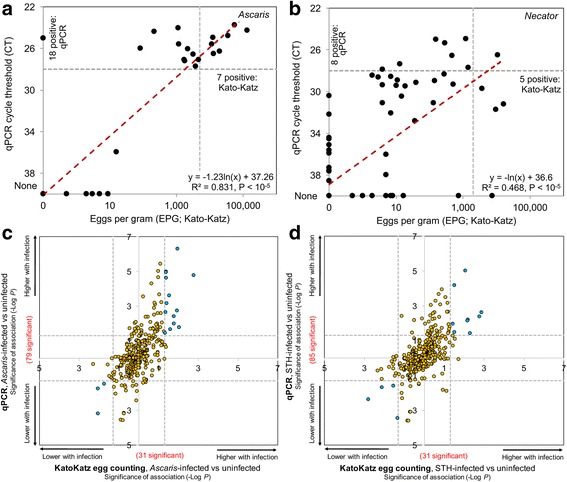
Comparisons of Kato-Katz and qPCR approaches for quantifying STH infections (based on Liberia samples). qPCR identifies more samples as being positive for *Ascaris* (**a**) and *Necator* (**b**), and identifies a different subset of samples as being infected than Kato-Katz quantification. Horizontal dashed lines = 28CT threshold for positivity, vertical dashed lines = moderate-infection according to Kato-Katz threshold, red dashed lines = line of best fit. **c**, **d** Significance values for the association of bacterial taxa with infections of *Ascaris* (**c**) and any STH (**d**). Blue dots = significant in both comparisons, dashed lines = adjusted significance thresholds of 0.05

Morbidity due to STHs is strongly correlated with infection intensity [29], as measured by the Kato-Katz smear in eggs per gram (EPG) of feces (per WHO recommendations). However, it is widely recognized that this microscopy-based test is suboptimal and labor insensitive [30], with high day-to-day variation [31]. In contrast, quantitative real-time PCR (qPCR) provides consistent results for detecting single or multiple parasitic infections in stool (mixed infections are common in most STH endemic countries [32, 33]). For the 98 samples collected from Liberia (Table 1, Additional file 1: Table S1), we compared STH abundance using both qPCR cycle threshold (CT) values (Additional file 4: Table S4) and the standard EPG procedure (Fig. 2a, b) for samples with “moderate/heavy infections” according to EPG thresholds recommended by the WHO (over 5000 and 2000 EPG for *Ascaris* and *Necator*, respectively [11, 34]). Consistent with a previous study analyzing *Ascaris* and *Necator* fecal abundance [32], a significant correlation between EPG and CT was found for both *Ascaris* (Fig. 2a) and *Necator* (Fig. 2b), the two most prevalent species among these individuals (*P* < 10^− 5^). However, at a CT value of 28 and below (chosen based on the lines of best fit for both *Ascaris* and *Necator*, as well as being the median detectable value in the previous study for *Necator* [32]), different subsets of individuals were identified as being moderately or heavily infected [35], with the qPCR method being more sensitive and identifying more individuals as STH positive overall. For all comparative analysis in this study (Fig. 1), only individuals with zero eggs and zero detection by qPCR (CT value ≥ 40) were used as negative controls, and individuals with ≤ 28 CT were considered infected.

The downstream effect of quantifying the level of infection with qPCR instead of egg counting on bacterial taxa associated with infection was tested using LEfSe [36], which performs both a non-parametric Kruskal-Wallis sum test and linear discriminant analysis to estimate effect size (Fig. 2c, d; Additional file 5: Table S5). Using this approach, 79 microbiome taxa were significantly associated with the *Ascaris*-infected individuals identified by qPCR (among samples from Liberia), whereas only 31 are significant when using the Kato-Katz identification method (Fig. 2c). Of the 18 taxa identified as significant by both approaches, 16 are less significant using the Kato-Katz identifications. Similarly, for infections with any STH (Fig. 2d), 54 more taxa are identified as significant by qPCR than by Kato-Katz (85 vs. 31, with 16 in common). Thus, the use of qPCR for STH quantification used here results in more sensitive detection of differentially abundant taxa, compared to microscopy-based approaches.

### Metadata analysis identified no significant cross-metadata associations

“Hierarchical All-against-All” (HAllA; http://huttenhower.sph.harvard.edu/halla) significance testing was used to identify significant associations between the various metadata, including infection status, cohort (according to definitions in Fig. 1), village, age, and sex. With the exception of positive *Ascaris* infections being associated with any positive infection in Liberia (18 of 26 infected samples had high *Ascaris* infections), no other significant associations were found between the various metadata according to thresholds used for HAllA in previous studies (FDR *q* < 0.05, similarity score > 0.5; [37]; Additional file 6: Figure S1A). Principal component analysis (PCA) was also used to cluster the Liberia and Indonesia samples according to relative taxa abundance values. For each of the metadata visualized (infection status in Additional file 6: Figure. S1B and S1D, and village and sex in Additional file 6: Figure. S1C and S1E), PERMANOVA [38] did not identify a significant clustering association (*P* > 0.1 in all comparisons), suggesting that it is not one of the metadata variables that is driving the overall microbiome profiles of the individuals in the study, and that infection status did not significantly correlate with any of the metadata.

### STH-associated gut microbiota defined and validated using independent geographical cohorts

**Fig. 3:**
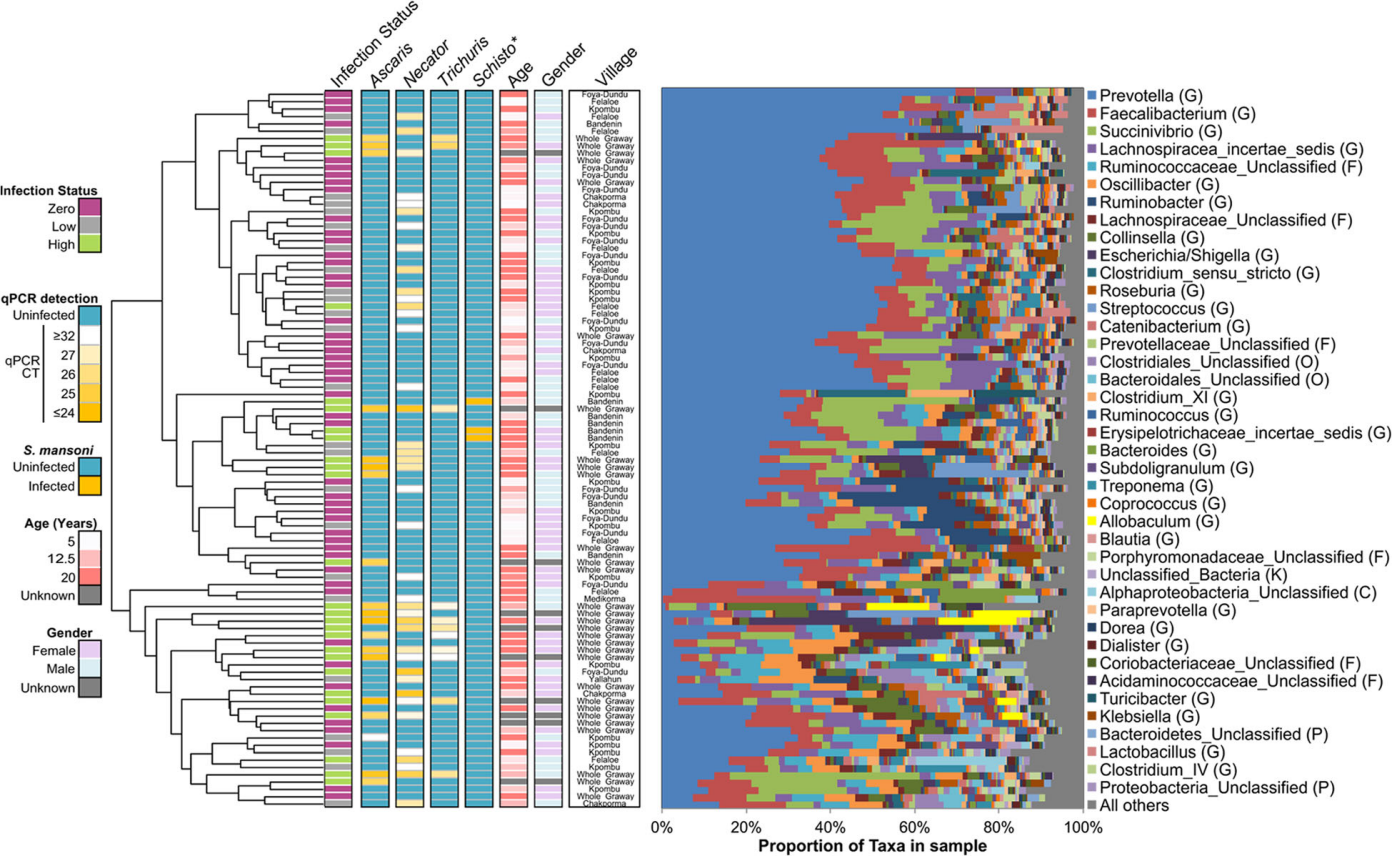
Bray-Curtis distance-based clustering of the 98 fecal samples from Liberia based on microbiota profiles

Of the 98 individuals sampled from Liberia, 26 had high STH infections (≤ 28 CT), while 48 had zero STH infection. *Prevotella* was dominant among the 98 individuals accounting for an average of 32% of all 16S reads (Fig. 3). Species richness was increased with STH infections (89.5 vs 82.1 with no infection; *P* = 5.8 × 10^− 3^, 2-tailed *t* test with unequal variance, Shapiro-Wilk test 0.98 for normal distribution) and Shannon diversity index (measures evenness and richness of communities within a sample) value was also increased (2.8 vs 2.4; *P* = 0.0008, Shapiro-Wilk test 0.96), indicating higher alpha diversity for infected individuals. In addition to an increased alpha diversity, the average between-individual diversity (Bray-Curtis beta diversity) was higher among the infected individuals (0.52) than among the uninfected individuals (0.43; *P* < 10^− 5^, 2-tailed *t* test with unequal variance; Shapiro-Wilk *W* = 0.978).

**Fig. 4:**
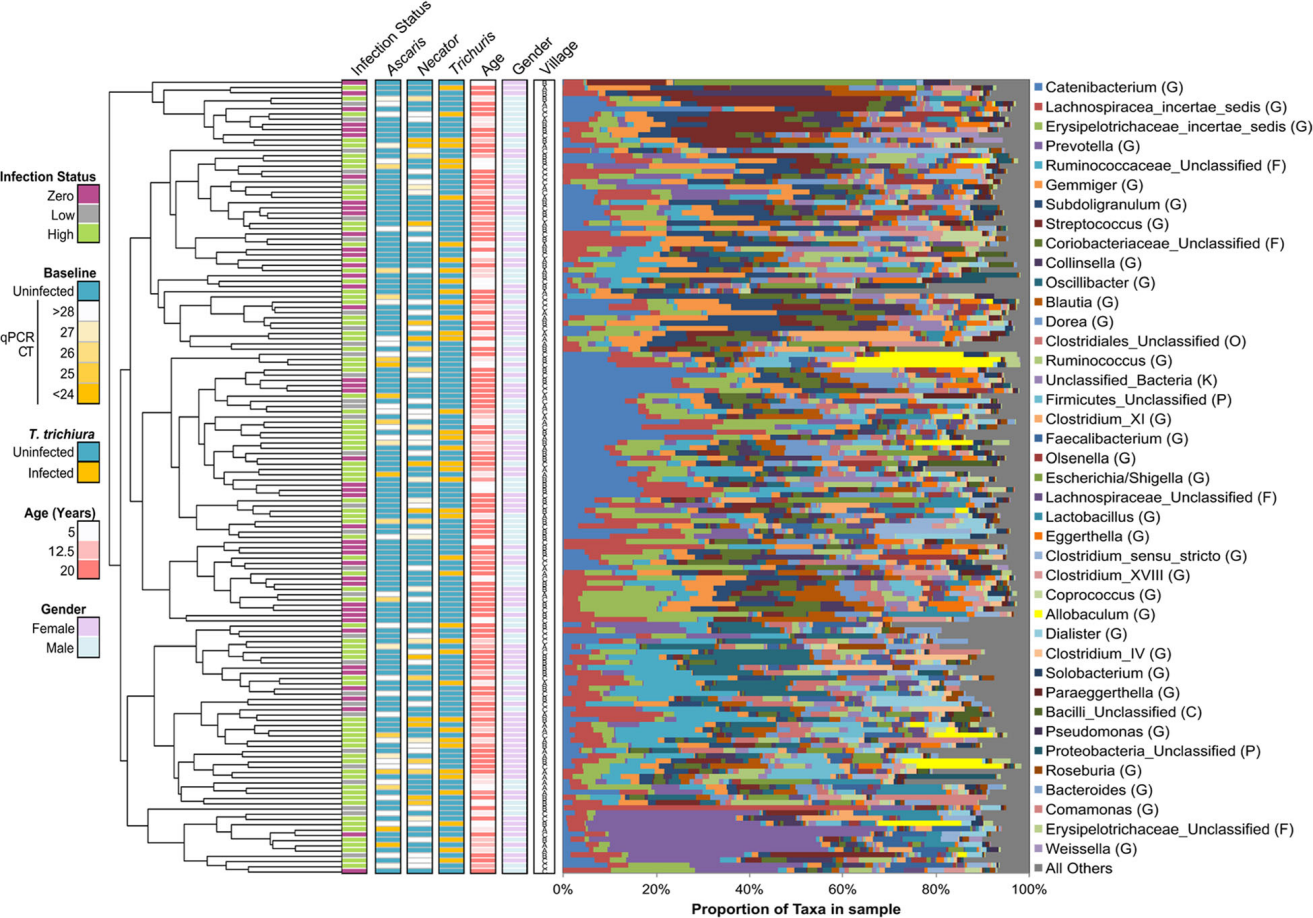
Bray-Curtis distance-based clustering of the 152 fecal samples from Indonesia (at baseline, 2008) based on microbiota profiles

As a cross-cohort baseline (pre-treatment) comparison, we also characterized the microbiome in a separate cohort comprised of 152 individuals from Indonesia (using the 2008 samples; Table 1, Additional file 2: Table S2). Unlike in Liberia, a wide variety of taxa dominate the microbiomes of individuals in Indonesia (Fig. 4), resulting in less structured clustering. In Indonesia, within-individual alpha diversity (Shannon diversity) was higher than in the Liberia individuals (average 3.0, no significant difference between infected and uninfected) and species richness was lower (partially due to the sequencing platform; Additional file 7: Figure S2). However, the pattern of higher richness among infected individuals was also observed here (66.9 in infected individuals, 63.8 in uninfected individuals; *P* = 0.049). Unlike in Liberia, the between-individual diversity among infected individuals in Indonesia (0.606) was not significantly different than among uninfected individuals (0.607; *P* = 0.86).

**Fig. 5:**
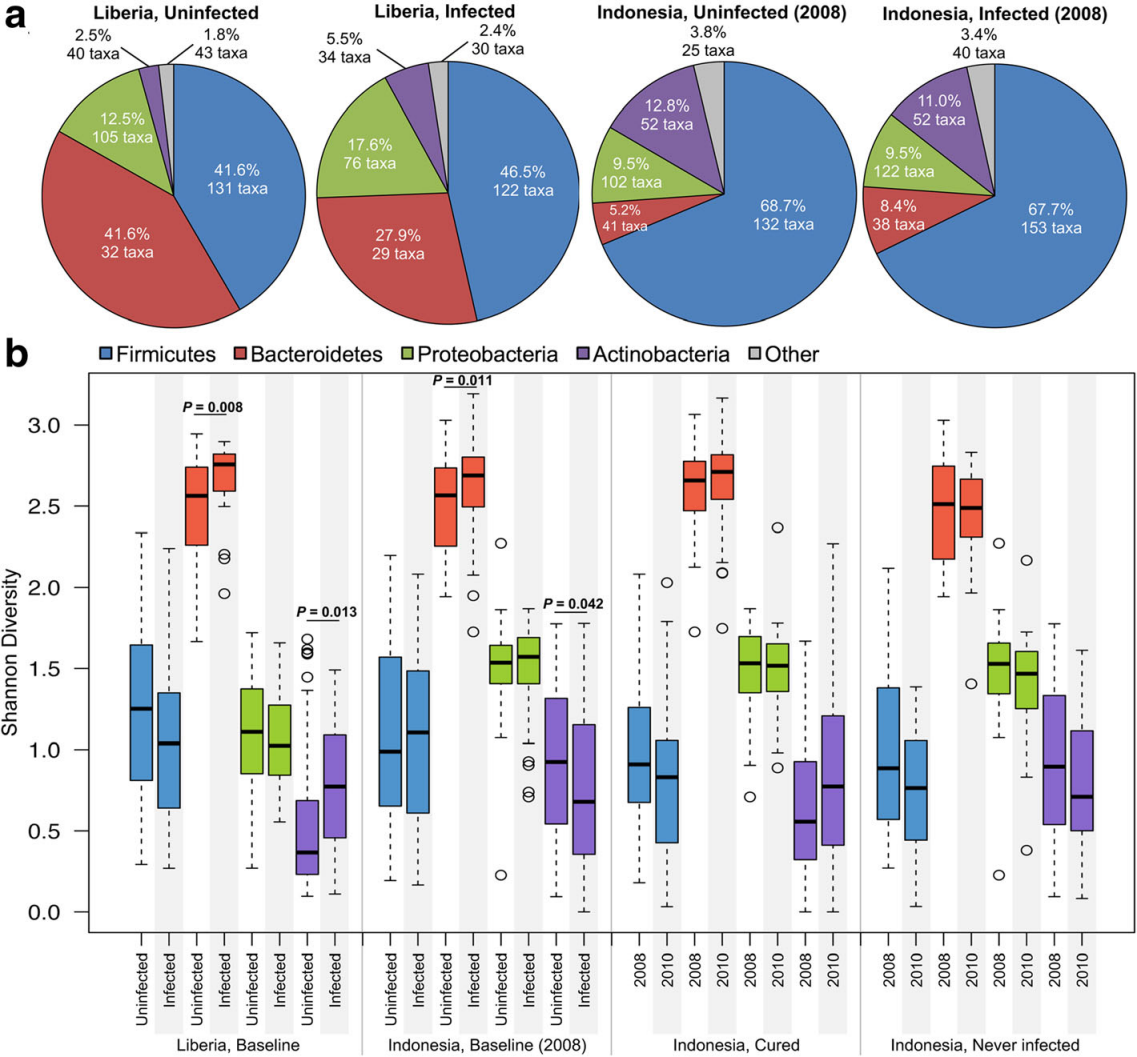
Phylum-level microbiome comparisons between Liberia and Indonesia, infected and uninfected individuals. **a** Relative phylum abundance and richness for each sample set. **b** Per-phylum comparisons of within-sample diversity among each sample set

Overall, between-individual diversity was much higher in Indonesia (0.618) than in Liberia (0.473; *P* < 10^− 5^), providing a distinct cohort for comparison. The overall gut microbiome compositions at the phylum level (Fig. 5) indicated that the baseline microbiomes for infected and uninfected individuals (Fig. 5a) in Indonesia were dominated by *Firmicutes*, while the Liberia individuals were dominated by a similar ratio of both *Firmicutes* and *Bacteroidetes*, with a reduction of *Bacteroidetes* among infected individuals. The dataset from the previously published Ecuador study [27] identified *Firmicutes* and *Bacteroidetes* abundances between those of Indonesia and Liberia, with no large differences in phylum abundance between infected and uninfected individuals (Additional file 8: Figure S3). Comparing the Shannon diversity index for each phyla between infected and uninfected individuals (Fig. 5b), we observed that *Bacteroidetes* had a significantly higher diversity among infected individuals in both countries (*P* = 0.008 in Liberia and *P* = 0.011 in Indonesia) while *Actinobacteria* diversity was higher among infected individuals in Liberia (*P* = 0.013) but lower among infected individuals in Indonesia (*P* = 0.042). These results indicate that different phyla dominate the samples from the different cohorts, and that the STH-associated differences in the overall phyla are not consistent between them.

**Fig. 6:**
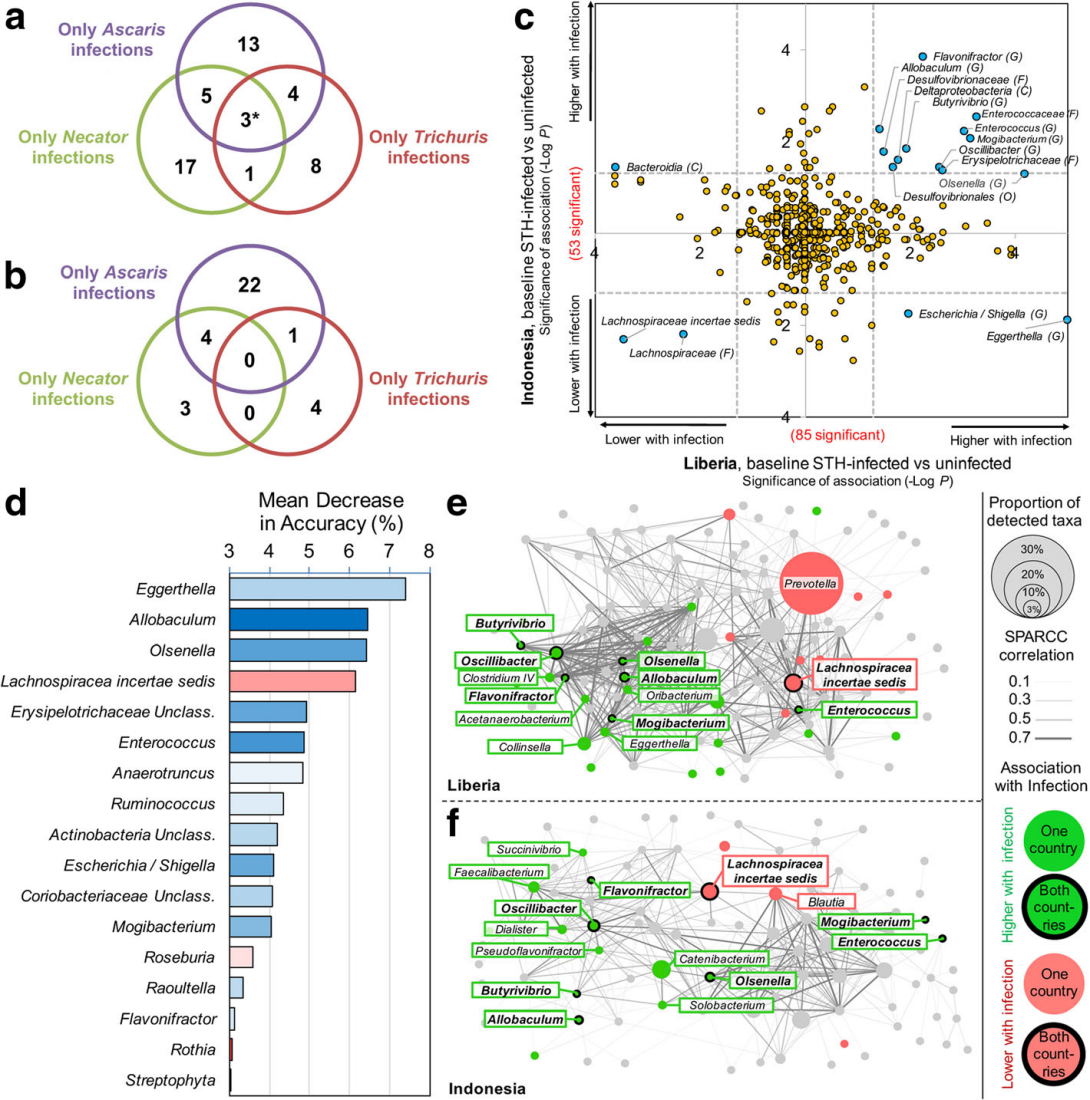
The identification of taxa significantly associated with helminth infection. Baseline (2008) positively (**a**) and negatively (**b**) STH-associated taxa are identified among individuals from Indonesia who were only infected by a single STH species. **c** Consistent STH associated-taxa are identified in both Liberia and Indonesia. **d** The STH-discriminatory taxa of highest importance in classifying samples from the random forest (RF) analysis. Only taxa with an “importance score” greater than 3 are shown in the figure, and many of these are the same taxa identified by LEfSe analysis in both countries. **e** A correlation network, connecting genera based on abundance values across samples from Liberia. **f** A correlation network, connecting genera based on abundance values across samples from Indonesia. Taxa of interest are identified with labels (bolded when significant in both countries only)

Differential bacterial taxa abundance was tested using LEfSe [36]. First, taxa associated with single STH infections (zero abundance of other STHs) were identified for individuals infected with *Ascaris* (*n* = 12), *Necator* (*n* = 12) or *Trichuris* (*n* = 19) in Indonesia (baseline 2008; Fig. 1, Fig. 6a, b). Most of the identified differentially abundant taxa (38/51 positively and 29/34 negatively associated) were associated with a specific STH species infection, while one genus (*Succinivibrio*) and its family and order were positively associated with infection of each of the STHs individually. *Solobacterium* was positively associated with *Necato*r and *Trichuris*, while *Desulfovibrio* and its family, order and class were positively associated with *Ascaris* and *Trichuris* (also previously found to be increased in pigs infected with *T. suis* [39])*.* Five taxa (four *Firmicutes* and one unclassified *Bacteroidetes*) including *Allobaculum* were positively associated with *Ascaris* and *Necator*. Additionally, four taxa including *Lachnospiraceae* (also discussed below) were negatively associated with *Ascaris* and *Necator*, and *Rhodococcus* was negatively associated with *Ascaris* and *Trichuris* (Additional file 5: Table S5). These results indicate that individual STH species mainly associate with specific microbiome taxa.

Comparisons of the taxa significantly associated with any STHs in both countries (Fig. 6c; Fig. 1; 2008 samples used for Indonesia) showed that *Lachnospiraceae incertae sedis* was the only consistently negatively associated genus, and the most significant positively associated genera were *Olsenella*, *Flavonifractor*, and *Enterococcus*. Altogether, 7 of the 12 taxa increased with infection in both countries belonged to the *Firmicutes* phylum, including 4 genera from the *Clostridales* order.

We have also independently analyzed the previously-published Ecuador dataset [27] (*n* = 97) using our analysis pipeline (data from the study on Malaysia individuals [26] was not available in public databases), identifying associations (positive or negative) between STH infections and 17 genera. All of the 7 individual taxa positively associated with STH infection in Ecuador belonged to the phylum *Firmicutes*, including 2 taxa from the class *Clostridia* (which included the genera Eubacteria) and 5 taxa from the class *Bacilli*, which included 3 *Lactobacillales* (order) taxa and the genera *Streptococcus*. In our current study, we likewise have identified cross-cohort STH-associated members of *Lactobacillales* (*Enterococcus*, *Enterococcaceae*, and an unclassified *Lactobacillaceae* family; *P* = 0.002 and 0.0003 for over-representation compared to all detected taxa in Liberia and Indonesia, respectively; binomial distribution test) and *Clostridia* (the genera *Butyrivibrio*, *Flavonifractor*, *Mogibacterium*, and *Oscillibacter*; not significantly over-represented compare to all detected taxa), with a total of 10 of the 15 cross-cohort taxa belonging to *Firmicutes* (*P* = 0.0004 and 0.02 for over-representation compared to all detected taxa in Liberia and Indonesia, respectively).

### Microbiome-based predictive STH-infection model and validation

We utilized a supervised machine-learning technique for identifying non-linear relationships in high dimensional microbiome data (random forest (RF) [40]) to identify STH-infection discriminating taxa in the Liberia individuals (Fig. 6d), and tested its predictive value in the Indonesia individuals. The RF model built using the Liberia samples had 74.3% classification power within the Liberia dataset (by “out of bag” error), and 72.4% predictive power for STH infections for the Indonesia validation dataset, with the top-ranked taxa being *Eggerthella*, *Allobaculum*, *Olsenella*, and *Lachnospiracae incerta sedis* (according to their “importance score”, measured using the mean decrease in the model’s accuracy as a result of excluding a taxa [1]). The LEfSe and RF approaches identify many of the same microbiota members as being significantly associated with STH infection in both geographical regions, including *Olsenella*, *Lachnospiracae incerta sedis*, *Allobaculum*, and other members of the *Erysipelotrichaceae* family, which provides further statistically independent verification of the cross-cohort association of these taxa with STH infection. As with the LEfSe analysis, most of the most significantly differentially abundant taxa had relatively low abundance in our dataset, highlighting the critical importance of both accurate and comprehensive sampling of the microbial communities. Performance improvement (up to 92% predictive value) can be achieved when phylogenetic dependency for grouping microbial data is used, as we have shown using subset of the individuals [41]; however, here a standard RF analysis is used for straightforward comparison to the LEfSe results.

### Abundance correlation networks identify clusters of infection-related taxa

In order to better understand the relationship between differentially abundant taxa, correlation networks were generated based on 16S abundances across all taxa identified at the genera level (Fig. 6e, f). SPARCC correlation values [42] (calculated based on 16S profiles while accounting for community diversity and utilizing appropriate statistics to deal with a high number of zero values) were utilized for network generation (*P* ≤ 0.05, correlation ≥ 0.2). Several genera were differentially abundant in both countries (Fig. 6e, f) and formed correlation subnetworks which include country-specific differentially abundant genera. In both countries, there are subnetworks connecting four consistently STH-associated genera (*Oscillibacter*, *Flavonifractor*, *Butyrivibrio*, and *Allobaculum*). In Liberia, this subnetwork includes *Mogibacterium* and *Olsenella*, but these taxa are separated in the Indonesia network. In both countries, *Enterococcus* is significantly associated with infection, but it clusters separately from the other taxa. These networks provide further insight into which consistent STH-associated taxa may be affected by similar biological mechanisms, or may possibly regulate each other.

### Gut microbiota respond to STH deworming and self-clearing

Longitudinal study in Indonesia was used to analyze changes in the microbiomes after deworming. Repeated measures of the same 152 individuals in 2008 and 2010 were compared. These individuals were classified into two study arms: uninfected individuals treated with anthelmintic or placebo, and infected individuals treated with anthelmintic or placebo (Fig. 1; Table 1 and Table 2; see “Methods”). Between the 2 years, 16 individuals were treated with anthelmintics but were never infected in either year with any STH species (Fig. 1; *treatment effect*), while another 16 were not treated and were also never infected in either year (*time effect*). Using the LEfSe enrichment approach (with individuals specified as a repeated-measures subclass to perform longitudinal analysis), we identified 10 taxa differentially abundant in the anthelmintic-treated but not the untreated uninfected individuals between the 2 years, including two genera-level taxa (Table 2 (A and B)): *Enterococcus*, which was higher in 2010 compared to 2008 only among treated individuals (*P* = 0.009), and *Ochrobactrum* (and its family *Brucellaceae*), which was lower in 2010 compared to 2008 only among treated uninfected individuals (*P* = 0.037; Additional file 3: Table S3). Earlier study tested 19 clinically relevant antimicrobial agents against *Ochrobactrum* isolates and showed their susceptibility to trimethoprim/sulfamethoxazole and ciprofloxacin [43] indicating that the anthelmintic albendazole may be having a similar effect as these antimicrobials.

**Table 2:** Significantly differentially abundant taxa between 2008 and 2010 among groups of individuals of interest from the Indonesia dataset

Phylum	Class	Order	Family	Genus	*P* value
A. Treatment effect—lower only after treatment					
*Proteobacteria*	*Alphaproteobacteria*	*Rhizobiales*	*Brucellaceae*		3.7E-02
*Proteobacteria*	*Alphaproteobacteria*	*Rhizobiales*	*Brucellaceae*	*Ochrobactrum*	3.7E-02
B. Treatment effect—higher only after treatment					
*Bacteroidetes*	*Sphingobacteria*				3.2E-04
*Bacteroidetes*	*Sphingobacteria*	*Sphingobacteriales*			2.0E-04
*Bacteroidetes*	*Sphingobacteria*	*Sphingobacteriales*	*Sphingobacteriaceae*		5.1E-04
*Bacteroidetes*	*Flavobacteria*				2.1E-03
*Bacteroidetes*	*Flavobacteria*	*Flavobacteriales*			2.1E-03
*Bacteroidetes*	*Flavobacteria*	*Flavobacteriales*	*Flavobacteriaceae*		2.1E-03
*Proteobacteria*	*Gammaproteobacteria*	*Pseudomonadales*			7.6E-03
*Firmicutes*	*Bacilli*	*Lactobacillales*	*Enterococcaceae*	Enterococcus*	9.3E-03
C. Deworming effect—higher only after deworming					
*Bacteroidetes*	*Sphingobacteria*	*Sphingobacteriales*	*Sphingobacteriaceae*	*Sphingobacterium*	3.7E-02
*Proteobacteria*	*Deltaproteobacteria*				3.8E-02
*Firmicutes*	*Erysipelotrichia*	*Erysipelotrichales*	*Erysipelotrichaceae*	Clostridium_XVIII**	4.2E-02
D. Deworming effect—lower only after deworming					
*Bacteroidetes*	*Bacteroidia*	*Bacteroidales*	*Prevotellaceae*	*Xylanibacter*	1.6E-02
*Proteobacteria*	*Betaproteobacteria*	*Burkholderiales*	*Sutterellaceae*		2.0E-02
*Firmicutes*	*Bacilli*	*Lactobacillales*	*Leuconostocaceae*	*Leuconostoc*	4.0E-02
*Bacteroidetes*	*Bacteroidia*	*Bacteroidales*	*Porphyromonadaceae*	*Butyricimonas*	4.2E-02
E. Self-cleared—higher only after self-clearing					
*Bacteroidetes*	*Bacteroidia*	*Bacteroidales*	*Porphyromonadaceae*		2.0E-02
*Bacteroidetes*	*Bacteroidia*	*Bacteroidales*	*Porphyromonadaceae*	*Parabacteroides*	8.5E-03
*Bacteroidetes*	*Bacteroidia*	*Bacteroidales*	*Bacteroidaceae*		2.7E-02
*Bacteroidetes*	*Bacteroidia*	*Bacteroidales*	*Bacteroidaceae*	*Bacteroides*	4.6E-02
*Synergistetes*					3.5E-02
*Synergistetes*	*Synergistia*				3.5E-02
*Synergistetes*	*Synergistia*	*Synergistales*			3.5E-02
E. Self-cleared—lower only after self-clearing					
*Actinobacteria*	*Actinobacteria*	*Coriobacteriales*	*Coriobacteriaceae*	Olsenella*	1.4E-02

*Also higher among infected individuals at baseline in Indonesia and Liberia (conserved STH-associated taxa)

**Also higher among uninfected individuals at baseline in Indonesia

A total of 27 STH-infected individuals were treated with albendazole and dewormed, resulting in no (zero) STH infection in 2010. Between 2008 and 2010, 18 taxa were differentially abundant for this group, 7 of which were not overlapping with the taxa from the treatment effect or time effect and were therefore considered to be differentially abundant as a result of deworming (Fig. 1; Table 2 (C and D)). Of these seven taxa, three were higher following deworming including *Sphingobacterium* (genus), an unclassified *Deltaproteobacteria* class, and *Clostridium_XVIII* (genus), and four were lower following deworming, including *Butyricimonas*. Overall, dewormed microbiomes in 2010 were more similar to the corresponding infected samples in 2008 (average Bray Curtis beta diversity = 0.630) than they were to uninfected samples in 2008 (beta diversity 0.642, *P* = 0.014), indicating that STH-associated microbiome taxa do not consistently restore back to baseline uninfected state following deworming.

As a test of whether microbiome state can predict long-term STH survival, we also compared the baseline (2008) untreated, infected individuals who remained infected in 2010 (*n* = 26) to those who were free from the infection in 2010 without anthelmintic treatment (“self-cleared”; *n* = 8; Fig. 1). We identified two taxa higher at baseline among those who maintained the infection (*Dialister* [genus], *P* = 0.048 and *Clostridium XIVa* [genus], *P* = 0.035) and 12 which were higher at baseline among those who would later self-clear, including the genus *Subdoligranulum* (*P* = 0.021; Additional file 5: Table S5). Looking between the 2 years, eight taxa were significantly differentially abundant among self-cleared individuals compared to de-wormed individuals and uninfected individuals (Table 2 (E and F)). The average between-sample diversity (Bray-Curtis beta diversity) was lower among the self-clearing individuals at baseline (2008) compared to individuals who were always infected (0.58 vs 0.61, *P* = 0.06), and this difference became higher and significant in 2010, (0.54 vs 0.60, *P* = 0.008), suggesting that self-clearing individuals had more alike microbiome structures compared to individuals who remained infected. Self-cleared individuals were significantly older on average (51.1 vs 21.4 years old; *P* = 0.012), and this trend was also significant in individuals in 2008 (*P* = 0.026), at which time uninfected people were an average of 30.8 years old and infected individuals were an average of 23.4 years old. This indicates that older individuals may be less exposed to reinfection or become more resistant to infection over time, and that specific microbiome taxa are either associated with or contribute to this resistance.

For the final differential 16S abundance test, we compared the pre-treatment microbiomes of treated individuals in Indonesia who remained infected (*N* = 11; non-responders) to those who were dewormed (*N* = 27; responders), in order to identify taxa which may facilitate or prevent worm clearance (as defined by a 28CT threshold for infection no detection for deworming). This test identified 7 taxa which were significantly associated with successful clearance following anthelmintic treatment, including the genera *Clostridium_IV*, *Turicibacter*, and *Collinsella*, along with all of its parent phylogeny (phylum *Actinobacteria*, class *Actinobacter*, order *Coriobacteriales*, family *Coriobacteriaceae*). In Liberia (but not Indonesia), *Turicibacter* was significantly negatively associated with baseline infection, suggesting that it may be preventative for infection and also help to clear infection (Additional file 5: Table S5). *Clostridium_IV* and *Collinsella* were both positively associated with infection in Liberia (but not Indonesia), so these genera are both associated with infection as well as with worm clearance after treatment. On the other hand, eight taxa were significantly associated with incomplete worm clearance or reinfection including the genus *Akkermansia* and its parent phylogeny, as well as *Ruminococcus* genus (and two of its parent phylogeny), which comprised and average 2.5% of the total microbiome in Indonesia and was significantly positively associated with infection in Indonesia. This identifies *Ruminococcus* as an important taxa both in terms of its association with infection at baseline and its prevention of parasite clearance.

### Species level diversity in helminth-associated microbiomes of newly infected and dewormed individuals

Metagenomic shotgun (MS) sequencing was performed to obtain the species level resolution of the microbiome from 24 stool samples (10 individuals, 2008 and 2010, from the Indonesia dataset and 4 individuals from the Liberia dataset; Additional file 3: Table S3). An average of 154 million MS reads were generated per sample and mapped to the bacterial reference genome database [44], identifying 618 unique bacterial taxa across the samples (average 301.4 per sample), with 82 taxa being identified across all 24 samples (the average taxa was identified in 11.7 of 24 samples). Based on the species-level Bray-Curtis diversity analysis, individuals who were both infected with any level of any STH infection (*n* = 19, average diversity 0.54) and individuals who both had zero STH infection (*n* = 5, average diversity 0.52) had more consistent within-group overall microbiome profiles than comparisons between the two groups (average diversity = 0.59; *P* = 0.011 compared to infected and *P* = 0.030 compared to uninfected). These results, with a relatively small sample set, provide further evidence for a distinct microbiome associated with helminth infection and provides (for the first time) strain level accuracy of the associated taxa.

### Helminth-associated genetic and metabolic potential of the gut microbial community

**Fig. 7:**
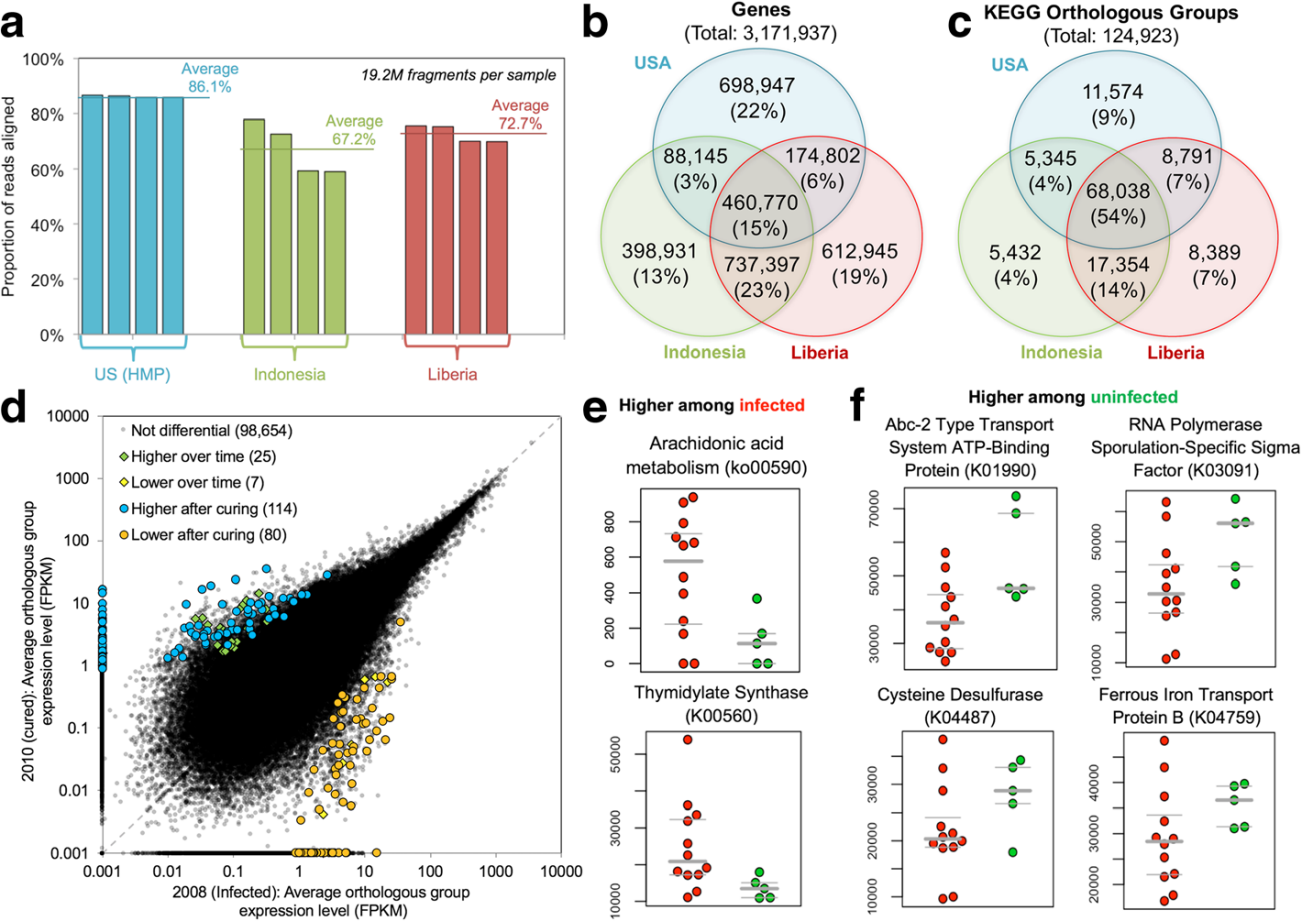
MS mapping rates per geographical region. **a** The proportion of reads mapped in each of four representative MS samples per country. **b** Distribution of genes identified among the four samples per country. **c** Distribution of functional KEGG OGs (KOGs) among the four samples per country. Although fewer genes are shared among the three regions, there was a much higher overlap in terms of functional potential. **d** Orthologous group differential expression between 2008 and 2010 for the four dewormed individuals from the Indonesia MS dataset. **e–f** Pathway and KOG abundance values between highly infected (red, *n* = 12) and uninfected (green, *n* = 5) individuals from the Indonesia MS dataset

The genetic potential of the microbial communities was evaluated by mapping the MS reads to a comprehensive gut bacteria reference gene catalog database (see “Methods”; obtained by stool samples from USA, Europe, and China [45]). In order to perform a direct comparison to samples from other regions, we used representative samples from the Indonesia dataset, along with representative samples from the USA (HMP data [44]) and from Liberia (Fig. 7; Additional file 4: Table S4), down-sampled to 37.8 million reads each. An average of 67.2 and 72.7% of Indonesia and Liberia reads (respectively) mapped to genes in the reference database, but this proportion of reads was much higher for the samples from the USA (86.1%; Fig. 7a), as expected due to its over-representation in the database. However, the overlap between Indonesia and Liberia was much higher (737,397 genes, 23% of all genes) than between either of these countries and the USA (88,145 genes and 174,802 genes, respectively). Only 46,770 genes (15% of detected genes) were conserved among the 3 countries (Fig. 7b), but an average of 45.7% of reads from each sample mapped to these genes. An average of 69.9% of functional groups per study area (68,038 KEGG OGs for all study areas) per sample was shared among the three sample groups (Fig. 7c). These results indicate that different gene sets perform similar functions between the three study areas.

In a separate analysis, reads from the 20 Indonesia samples were mapped to the integrated gene catalog (IGC) database [45]), and abundance values were compared per orthologous group (OG) before (2008) and after (2010) deworming using DESeq2 [46] (paired differential analysis). A total of 114 and 80 OGs were identified as statistically higher and lower after deworming (respectively) but not differential over time among consistently infected individuals (Fig. 7d; Additional file 3: Table S3). In addition, HUMAnN [47] was used to reconstruct the metabolic pathways of the 20 Indonesia shotgun metagenomic datasets, and pathway enrichment was performed using LEfSe [36] to compare STH-infected vs uninfected samples, using the same criteria as the 16S samples (total 12 STH-infected and 5 uninfected, with 3 low-infected samples excluded; Additional file 3: Table S3). The only significantly enriched KEGG pathway among the metagenomes of STH-infected samples was “arachidonic acid metabolism” (ko0059; Fig. 7e). The only KEGG Orthology (KO) category significantly enriched among the microbiomes of STH-infected individuals was “thymidylate synthase” (K00560). A total of 11 KOs were significantly depleted among STH-infected individuals (Table 3; top four visualized in Fig. 7f). These included (i) “RNA Polymerase Sporulation-Specific Sigma Factor” (K03091) and “Spore Coat Protein JC” (K06334), as well as “Cysteine Desulfurase” (K04487) and “Ferrous Iron Transport Protein B” (K04759). Together, these functional enrichment approaches identify many STH-associated microbiome functional pathways which offer further insight into these associations.

**Table 3:** KEGG pathways and Kos enriched among infected and uninfected MS samples

Category	Description	Identifier	Effect size	*P* value	Relative abundance	
					Infected	Uninfected
Higher among STH-infected individuals						
KEGG pathway	Arachidonic acid metabolism	ko00590	2.384	0.044	499.4	130.1
KEGG Orthology	Thymidylate synthase	K00560	2.286	0.035	25.0	13.7
Lower among STH-infected individuals						
KEGG pathway	(None)					
KEGG Orthology	Abc-2 type transport system ATP-binding protein	K01990	2.595	0.027	37.6	55.8
	RNA polymerase sporulation-specific sigma factor	K03091	2.582	0.045	35.0	50.9
	Cysteine desulfurase	K04487	2.206	0.045	21.8	28.1
	Ferrous iron transport protein B	K04759	2.199	0.035	29.2	35.6
	Putative hydrolases of HD superfamily	K07023	2.153	0.035	11.0	17.1
	Uncharacterized protein	K06950	2.146	0.045	32.2	38.2
	Aminotransferase	K10907	2.115	0.020	12.9	18.4
	Nad-dependent deacetylase	K12410	2.107	0.045	21.6	27.6
	mRNA interferase	K07171	2.081	0.011	15.5	20.8
	Spore coat protein JC	K06334	2.065	0.045	5.5	10.7
	Tryptophan synthase beta chain	K06001	2.048	0.020	5.8	11.1

## Discussion

We focused on identifying and validating biologically meaningful associations between STH infections and the gut microbiota and its functional potential by (a) comparing the fecal microbiome of moderately/heavily STH- infected individuals to uninfected individuals (detected by qPCR for increased sensitivity), (b) validating the findings using subjects from a distinct geographical cohort, (c) identifying conserved STH-associated and STH-discriminatory taxa, (d) delineating microbiome assemblage changes as a result of de-worming/self-clearing, and (e) identifying microbiome-encoded biological functions associated with STH-infected individuals. While answering these questions using several comparisons between multiple cohorts (Fig. 1), technical aspects were also considered to understand how study design, diagnostic accuracy, precise STH burden estimation, and depth of coverage affect optimal profiling of the microbiome.

Compared to Liberia which was dominated by *Prevotella*, the microbiomes from Indonesia individuals were comparatively highly variable and diverse, providing a distinct microbiome background for cross-cohort comparison. Comparisons of the specific taxa significantly associated with STHs in both countries (Fig. 6c) showed that *Lachnospiraceae incertae sedis* was the only consistently negatively associated genus, and the most significant positively associated genera were *Olsenella* (previously associated with reduced gut inflammation and multiple sclerosis symptoms when administered as a probiotic [48] and associated with lean individuals compared to obese individuals [49]), *Flavonifractor* (increased in the gut microbiome with systemic lupus erythematosus [50]), and *Enterococcus* (previously found to be increased in cats infected with *Toxocara cati* [51]). Another significant positively associated genus, *Allobaculum*, has been previously associated with gut inflammation [52] (with an elimination of this genera in mouse gut models with high inflammation [53]), and has been associated with weight reduction and negatively correlated with leptin and the inflammation marker *Slc25a25* in mice, suggesting an important role in host energy balance and intestinal responses [54].

Altogether, 7 of the 12 taxa increased with infection in both countries belonged to the *Firmicutes* phylum, including four genera from the *Clostridales* order. In our independent analysis of the previously published Ecuador dataset [27], all 7 individual taxa positively associated with STH infection in Ecuador belonged to the phylum *Firmicutes*, highlighting the association of this phylum with helminth infection. Most notably, the family *Lachnospiraceae* was significantly lower in infected samples in Ecuador (*P* = 0.037), and this was also one of just two taxa significantly lower in infected individuals in both Liberia and Indonesia (Fig. 4b). *Lachnospiraceae*, a common gut bacteria, has previously been linked to obesity and protection from cancer (due to its production of butyric acid, important for both microbial and host epithelial cell growth [55]) and was previously associated with modulating inflammation during the blood-feeding nematode *Haemonchus contortus* infections [24]. Thus, we identify the robust existence of STH-associated taxa that are consistent across broad geographical regions representing Africa, Asia, and America. In future research, the results may be further improved by using consistent techniques/parameters for infection quantification, cut-offs for infection level, matching controls, hypervariable rRNA gene region, and sequencing platform.

The LEfSe and RF approaches identify many of the same microbiota members as being significantly positively associated with STH infection in both geographical regions and with being discriminatory in the RF analysis, including: *Olsenella*, *Allobaculum*, and other members of *Allobaculum*’s family *Erysipelotrichaceae*, including positive associations with *Solobacterium* (negatively correlated with the production of connexin-43, involved in intestinal repair). This family has been found to be negatively associated with the flatworm *Opisthorchis viverrini* infection in hamsters [56], but here we identify both positive and negative associations with different genera from the family. Both *Olsenella* and *Allobaculum* have been previously associated with a reduction in gut inflammation [44, 53], suggesting that their association with STH infection may potentially have positive side effects on the host gut health. In the present study, for the first time, we demonstrate consistent STH-associated microbiome taxa across distinct geographical regions that helped us to successfully predict infectious status based on microbiome structure. In future studies, the predictive value of this model can be improved by increasing power obtained via sampling larger cohorts with a higher number of individuals harboring single-species infections and multiple-species infections. In addition, the existence of potentially functionally related taxa associated with STH infection (Fig. 6e, f) provides a precedent for further efforts focused on experimental testing synthetic communities of limited size.

Three taxa were higher as a result of deworming including *Clostridium_XVIII* (genus), which was also significantly less abundant in infected individuals at the 2008 baseline in Indonesia (*P* = 0.03) and less abundant, but not significantly (*P* = 0.08), in Liberia, indicating that this genus is both higher in infected individuals and reduced after deworming (Additional file 5: Table S5), making it an interesting candidate for future study. Four additional taxa were lower following deworming, including *Butyricimonas*, which is a butyrate-producing bacteria and butyrate is a potential inhibitor of inflammation, as discussed in the previous *H. contortus* microbiome study in goats [24]. Overall, microbiome assemblages of dewormed individuals still more closely resemble their corresponding infected status than the microbiome assemblages of the uninfected individuals. This is in contrast with the mice whipworm *T. suis* studies where clearance of infection resulted in gradual transitioning of the microbiome in an uninfected state [57]. Among self-cleared individuals (i.e., “dewormed” but without anthelmintic treatment), 8 taxa were significantly differentially abundant compared to de-wormed individuals and uninfected individuals (Table 2(E and F)), most notably *Olsenella*, which was significantly reduced over time in individuals who self-cleared (*P* = 0.014) and was the most significantly positively associated with infection in both Liberia and Indonesia, providing further evidence of this genus’ important association with STH infection.

Metagenomic shotgun (MS) sequencing was performed to obtain the species level resolution of the microbiome from 24 stool samples and provides (for the first time) strain level accuracy of the associated taxa. Genetic potential of the microbial communities was evaluated by mapping the MS reads to a comprehensive gut bacteria reference gene catalog database for representative samples from Liberia, Indonesia, and the USA (Fig. 7b), and this analysis suggested that different gene sets perform similar functions between the three countries, with a stronger conservation between Liberia and Indonesia. The read sequence data generated by this study provides a valuable resource for characterizing novel bacterial strains and biological functions unique to these resource-poor and under-sampled regions of the world.

In a separate analysis of the MS data against the Integrated Gene Catalog (IGC) OG differential expression, 114 and 80 OGs were identified as statistically higher and lower after deworming, respectively, but not differential over time among infected individuals (Fig. 7d; Additional file 3: Table S3). The OGs most significantly higher after deworming included “monovalent cation H antiporter subunit F” (possibly involved with drug transport [58]), and the most significantly lower after deworming (higher during infection) included “Macrocin-O-methyltransferase” (involved in the synthesis of an antibiotic that targets gram positive bacteria [59]). Interestingly, different “Fimbrial protein” OGs were significantly both higher and lower after curing, suggesting a consistent change in the types of fimbrial proteins, rather than an increase or decrease.

Only one previous study [26] has examined the metabolic potential of the fecal microbiome of STH-infected and uninfected individuals (identifying whipworm a primary driver of the overall differences), but this was not based on MS data, but rather on predicted microbial functions through inference from 16S-based microbiota profiling (using PICRUSt [60]). Here, HUMAnN [47] was used to reconstruct the metabolic pathways of the 20 Indonesia MS datasets, and pathway enrichment was performed. The only significantly enriched KEGG pathway among the metagenomes of STH-infected samples was “arachidonic acid metabolism” (ko0059; Fig. 7e). Arachidonic acid is the precursor for pro-inflammatory leukotrienes that threaten helminth survival, and previous studies have shown that a wide range of helminth species secrete products to modulate arachidonic acid and leukotriene activity [61], and arachidonic acid has also been successful as an anthelmintic treatment for *Schistosoma* infections in mice [62]. Here, we present the first evidence that some modulation of arachidonic acid activity in the STH-infected intestine may occur through the increase of arachidonic acid metabolizing bacteria.

The only KEGG Orthology (KO) category significantly enriched among the microbiomes of STH-infected individuals was “thymidylate synthase” (K00560), an essential enzyme for DNA synthesis with no clear function for supporting helminth infection. A total of 11 KOs were significantly depleted among STH-infected individuals (Table 3; top four visualized in Fig. 7f). These included (i) “RNA Polymerase Sporulation-Specific Sigma Factor” (K03091) and “spore coat protein JC” (K06334), suggesting a reduction in spore-forming bacteria in association with STH infections, as well as “cysteine desulfurase” (K04487) and “ferrous iron transport protein B” (K04759), both of which are important for cellular iron homeostasis [63, 64]. These categories may be reduced due to more readily available iron from blood in the intestine, released while the hookworm attaches on the lining of the intestinal wall to feed on blood. Overall, the MS analysis offers a more detailed view of specific microbiome-encoded functions which are associated with STH infection.

## Conclusions

Specific microbiome assemblages are significantly associated with STH infection, and specific members of the gut microbiome discriminate between moderately/heavily STH-infected and non-infected states across very diverse geographical regions using two different statistical methods. Microbiome-encoded biological functions associated with the STH infections were identified, which are associated with STH survival strategies and changes in the host environment. These results provide a novel insight of the cross-kingdom interactions in the human gut ecosystem by revealing STH-associated microbiome assemblages at taxonomic, genetic, and functional levels, so that advances towards key mechanistic studies can be made.

## Methods

### Study design and cohorts

For samples from Liberia, a single stool sample was collected from individuals living in remote areas of north-western Liberia (Foya District, Lofa County; *N* = 68) or in coastal eastern Liberia (Harper District, Maryland County; *N* = 30). Stool samples and demographic information were collected as part of a larger survey on the long-term impact of mass drug administration (MDA) to eliminate lymphatic filariasis and onchocerciasis. While Foya was co-endemic for intestinal schistosomiasis, Harper was not. No previous de-worming was conducted in the study areas for at least 6 to 8 months before sample collection, but areas had previously received one or two rounds of MDA with ivermectin plus albendazole. Labeled stool containers were handed out in the evening and were collected early next morning. Within 6 h of sample collection, Kato-Katz smears were prepared and 1 g of stool was preserved in 4 mL RNAlater (Ambion) and stored at 4 °C until DNA extraction, or frozen at − 20 °C for long-term preservation.

The sample collection and processing procedure for the samples from Indonesia have been previously described [65]. In short, stool samples were collected before and after anthelminthic treatment. *T. Trichiura* infection was detected by microscopy using the formol ether concentration method, while a multiplex real-time PCR was used for detection of hookworm (*Ancylostoma duodenale*, *Necator americanus*), *Ascaris lumbricoides* and *Strongyloides stercoralis* DNA. For the current study, paired DNA samples before and after treatment (21 months) from 152 inhabitants in Nangapanda were selected based on the anthelmintic treatment allocation and infection status, as well as the availability of complete stool data at both pre and post-treatment time points.

No subjects with acute infections or other acute illnesses were included in the study as they would be not eligible for anthelminthic mass drug administration. This excludes also subjects with acute malaria. In these rural settings, the HIV prevalence is generally low. Furthermore, mapping of our shotgun metagenomic reads to a database of lower eukaryotes did not show presence of specific non-worm infections. While we have not tested all subjects for specific protozoan, viral, or bacterial infections, we control for this by doing a randomized trial. For this study we postulated that any subclinical infections were evenly distributed between the study groups and did not affect clustering.

### Helminth detection using the Kato-Katz procedure and qPCR analysis

In Liberia, duplicate Kato-Katz smears were prepared to estimate the number of helminth eggs per gram of stool as recommended by WHO [66]. Each smear contained 41 mg of fresh stool and was examined 30 to 90 min after preparation to ensure the integrity of the hookworm eggs. For detection of helminth DNA by qPCR, DNA was extracted from 300 μl of stool/RNAlater suspension containing approximately 60 mg of feces. RNAlater was removed by high-speed centrifugation, and the sample’s pellet was homogenized in ASL buffer (Qiagen, Hilden Germany) using a Precellys 24 homogenizer (Bertin Technologies, France) and the Precellys Lysing Kit for soil grinding (Bertin Technologies, France). Then, the homogenate was boiled for 10 min and DNA was extracted using the DNeasy Blood and Tissue Kit (Qiagen, Hilden, Germany) according to the instructions of the manufacturer. The DNA content of the extractions was measured using a Qubit fluorometer (Invitrogen, Carlsbad, CA). Usually, a DNA concentration between 200 and 500 ng/μl was achieved per extraction and was required for the samples to be suitable for further study.

The collection and processing procedures for the samples from Indonesia have been previously described [67]. Parallel qPCR reactions to detect DNA of *Ascaris*, *Necator*, *Trichuris*, *A. duodenale*, and *S. mansoni* were performed using 5 ng total DNA template per 10 μl reaction volume and a QuantStudioFlex6 thermocycler (Applied Biosystems; Additional file 4: Table S4). No *A. duodenale* DNA was detected in either of the study sites, while *S. mansoni* was only detected in three samples in Foya (considered STH-positive in the analysis). Although samples from Indonesia and Liberia were tested using different types of thermocyclers and slightly different qPCR assays, the sensitivity of qPCR for both countries was similar. In this study, for both countries, qPCR detection at a CT value of 28 or lower was used to identify “moderately/heavily” STH-infected samples. Samples with a positive qPCR identification, but with greater than 28CT detection were identified as “low”-level infections (and not included in the analysis), and those with no detection by qPCR were considered uninfected. As shown in Fig. 2a, the *Ascaris* line of best fit (between egg count and qPCR CT) cross the WHO threshold of 5000 EPG at a value of 26.8, and the *Necator* line of best fit (Fig. 2b) crosses its 2000 EPG threshold at a value of 29. The average of these two values 27.9, which was rounded up to 28, and is consistent with the median detectable value in the previous study for *Necator* [32]*.*

### 16S rRNA gene sequencing and microbial community characterization

The V1–V3 hypervariable region of the 16S rRNA gene was amplified by PCR using the 27F and 534R primers “AGAGTTTGATCMTGGCTCAG” and “ATTACCGCGGCTGCTGG.” The PCR products from the Liberia samples were purified and sequenced on the MiSeq Genome Sequencer generating, on average, 24,000 reads per sample (Illumina, San Diego, CA). The Indonesia PCR products were sequenced on the Genome Sequencer Titanium FLX (Roche Diagnostics, Indianapolis, IN), generating an average of 6000 reads per sample (differences in the depth of coverage are likely to partially affect the cross-cohort comparisons)*.* Raw sample reads are accessible for download from NCBI’s Sequence Read Archive (SRA, BioProject #PRJNA407815; *Upload in progress*) and from Nematode.net (Nematode.net/Microbiome.html). A subset of the Liberia samples were also sequenced using the 454/Roche sequencer (using the same variable region), to facilitate a comparison of the sequencing platforms. The degree of overlap for the genera captured by the MiSeq and the Titanium FLX are shown in Additional file 8: Figure S3A. The average reads per taxa obtained with the V1–V3 primers are provided in Additional file 8: Figure S3A.

Sample sequences were binned based on Illumina index sequences and by removing their tags in flow space (Roche Diagnostics), with one mismatch allowed. Paired end fastq files were generated and the primers were removed from the 3′ end of the sequence using Trimmomatic [68] and Flexbar [69], allowing 1 mismatch in addition to primer degeneracies. Low quality bases were removed using Mothur software [70] with the parameter “trim.seqs” (qaverage = 35). Illumina paired reads were assembled using FLASH [71] and then assembled. For the Roche Diagnostics sequences less than 200 bases were removed. Taxonomic calls were generated for each assembly and Roche Diagnostics reads using the Ribosomal Database Project Naïve Bayesian Classifier (version 2.5 with training set 9 [72], as previously performed; e.g., [73]). Chimeric sequences were identified and removed using ChimeraSlayer, with default parameters [74]. To analyze the diversity at various taxonomic levels, RDP-generated taxonomic calls were analyzed to generate sample vs. taxonomy matrices, where a 0.5 confidence level was required to accept a call at each taxonomic level. For example, reads with < 0.5 confidence at the genus level were considered “unclassified” at the family level.

### Shotgun metagenomic sequencing and microbiome genetic potential profiling

Shotgun metagenomic libraries were generated and sequenced on the Illumina HiSeq2000 platform. Raw sample reads are accessible for download from NCBI’s Sequence Read Archive (SRA, BioProject #PRJNA407815; *Upload in progress*). An average of 154 million whole genome shotgun reads were captured from metagenomic DNA collected from Indonesia fecal samples (*n* = 20) from 10 individuals (sampled in both 2008 and 2010), as well as four Liberia individuals. Illumina genomic DNA sequence for the 24 samples were retrieved from the Laboratory Information Management System database and subject to human contaminant screening using BMTagger (version 1.0) as previously described. The non-human reads were then filtered to remove redundancy (using the Picard’s Estimate Library Complexity method, release 1.27), low quality reads were trimmed using the TrimBWAStyle.pl script (which applies the quality trimming logic used by Burrows-Wheeler Aligner) and low complexity reads (as detected by the DUST program) were removed. DUST masks low quality sequence that it identifies and reads were discarded anytime fewer than 60 unmasked bases remained after applying DUST. MS RNA-Seq reads were mapped to the HMP bacterial database).

Cleaned reads from the 20 Indonesia samples were downsampled to ~ 142 million reads (~ 69 paired end + ~ 3.8 M orphans) for equal comparisons between sample sets. These were then mapped to the integrated gene catalog (IGC) database) using bowtie2.2.5 (default parameters). Coverage was calculated using the bedtools coverage utility (v2.17.0). For functional comparisons, IGC genes were annotated using eggnog. Genes were considered “identified” if they had a minimum of five reads mapped from a minimum of three of samples. Gene length-normalized abundance values were then averaged per orthologous group (OG). OG abundance values (multiplied by 1000 to account for normalization) were then used as input for DESeq2 differential expression analysis (default settings), to identify genes more highly abundant before (2008) and after (2010) deworming among the four individuals who were successfully dewormed of heavy infections (paired differential analysis; Additional file 3: Table S3).

The cleaned reads were also mapped to the Kyoto Encyclopedia of Genes and Genomes (KEGG) genes database (v58) [75, 76] using the MBLASTX program with default parameters [77]. Results of this mapping were used as input to The Human Microbiome Project Unified Metabolic Analysis Network program (HUMAnN) [47] to obtain pathway and KEGG identifier abundances. These abundances were finally fed into the LEfSe [36] and were used to calculate enriched pathways and/or KEGG identifiers between samples infected with each of three STH species for which there was a sufficient number of infected and uninfected samples (*Necator* and *Trichuris*). The qPCR identifications of helminth abundance were used to classify the infection status of samples to be consistent with other analyses.

### Differential microbiome abundance

LEfSe [36] was used for differential taxa abundance testing, using default recommended settings according to the author’s instructions, at an adjusted *P* ≤ 0.05 for significance and requiring an LDA effect size of at least 2 for every significant call. LEfSe’s algorithm performs class comparison tests and validates for biological consistency, and is able to consider all taxonomic levels for comparison simultaneously. Many studies have utilized LEfSe for microbiome comparison testing to identify gut microbiome members associated with pathogenic infections [78] to identify gut biomarkers for disease [36] and to track microbiome recovery following disease [79].

### SPARCC correlation network analysis

For Liberia and Indonesia 16S sample sets individually, abundance values for all taxa identified to the genus level in a minimum of two samples (normalized to the total mapped read count per sample) were used as input for SPARCC correlation analysis [42] (default settings). SPARCC correlation values were specifically designed for the challenging task of identifying meaningful significant associations among 16S profiling datasets, accounting for community diversity and utilizing appropriate statistics to deal with a high number of zero values [42]. For the final network visualization, a minimum correlation of 0.2 and a minimum significance of association *P* ≤ 0.05 was required for inclusion (Fig. 6e, f; Additional file 3: Table S3). Networks were visualized using Cytoscape [80] (version 3.3.0) using the “edge weighted force directed layout” based on the correlation values. Identifications of differentially abundant taxa were based on identifications from the “differential microbiome abundance” subsection (above).

### Random forest analysis

We used random forest (RF), a supervised machine-learning technique [40], to identify the bacterial genera that differentiate STH-infected and STH-uninfected microbiomes (according to qPCR standards). RF can identify non-linear relationships from high-dimensional and dependent data, and it is especially suitable for microbiome datasets [40]. Using samples from Indonesia, we built an RF model using 10,000 trees and default parameters are applied for model construction. The generalization error of the model was evaluated by out of bag (OOB) error. To test the prediction accuracy of the model, Liberia samples with the same 37 genera served as the validation set. The prediction power of the model was evaluated using the receiver operating characteristic (ROC) analysis. The genera that are highly predictive of moderate to heavy infection and non-infection were identified using importance score. An importance score of at least 0.001 was considered highly predictive, as previously reported [1].

## Additional file

Additional file 1: Table S1. Metadata and read counts per taxa for the 98 16S samples from Liberia. (XLSX 595 kb)

Additional file 2: Table S2. Metadata and read counts per taxa for the 216 16S samples from Indonesia. (XLSX 1655 kb)

Additional file 3: Table S3. Enrichment results, metadata and depths of coverage per genome for the 24 MS samples from Indonesia and Liberia. (XLSX 221 kb)

Additional file 4: Table S4. qPCR primers used to test for STH presence (XLSX 39 kb)

Additional file 5: Table S5. Enrichment of bacterial taxa among *Ascaris* and helminth-infected samples, according to LEfSe results. (XLSX 788 kb)

Additional file 6: Figure S1. Metadata analysis for Indonesia and Liberia datasets. (A) Hierarchical All-against-All (HAIIA) significance testing for metadata. (B–E) Principal component analysis (PCA) plots based on relative taxa abundance for all taxa identified in three or more samples, among heavy-infected or non-infected samples, are shown for Indonesia, with color coding according to (B) comparison cohort and (C) village and sex metadata, and for Liberia, with color coding according to (D) infected vs uninfected samples and (E) village and sex metadata. No significant differential clustering was identified, according to PERMANOVA. (TIFF 429 kb)

Additional file 7: Figure S2. Comparison of MiSeq and 454 sequencing platforms. (A) MiSeq identifies more unique bacterial taxa to the genus and family than 454. (B) Identified taxa are supported by more reads with MiSeq than with 454, improving statistical comparison power. (TIFF 334 kb)

Additional file 8: Figure S3. Relative phylum abundance and taxa counts for the Ecuador sample set [27]. (TIFF 146 kb)
