# Supplementary figures and images for: Differential human gut microbiome assemblages during soil-transmitted helminth infections in Indonesia and Liberia
Source: Microbiome. 2018 Feb 28;6:33. doi: 10.1186/s40168-018-0416-5 (PMC6389212; doi:10.1186/s40168-018-0416-5)

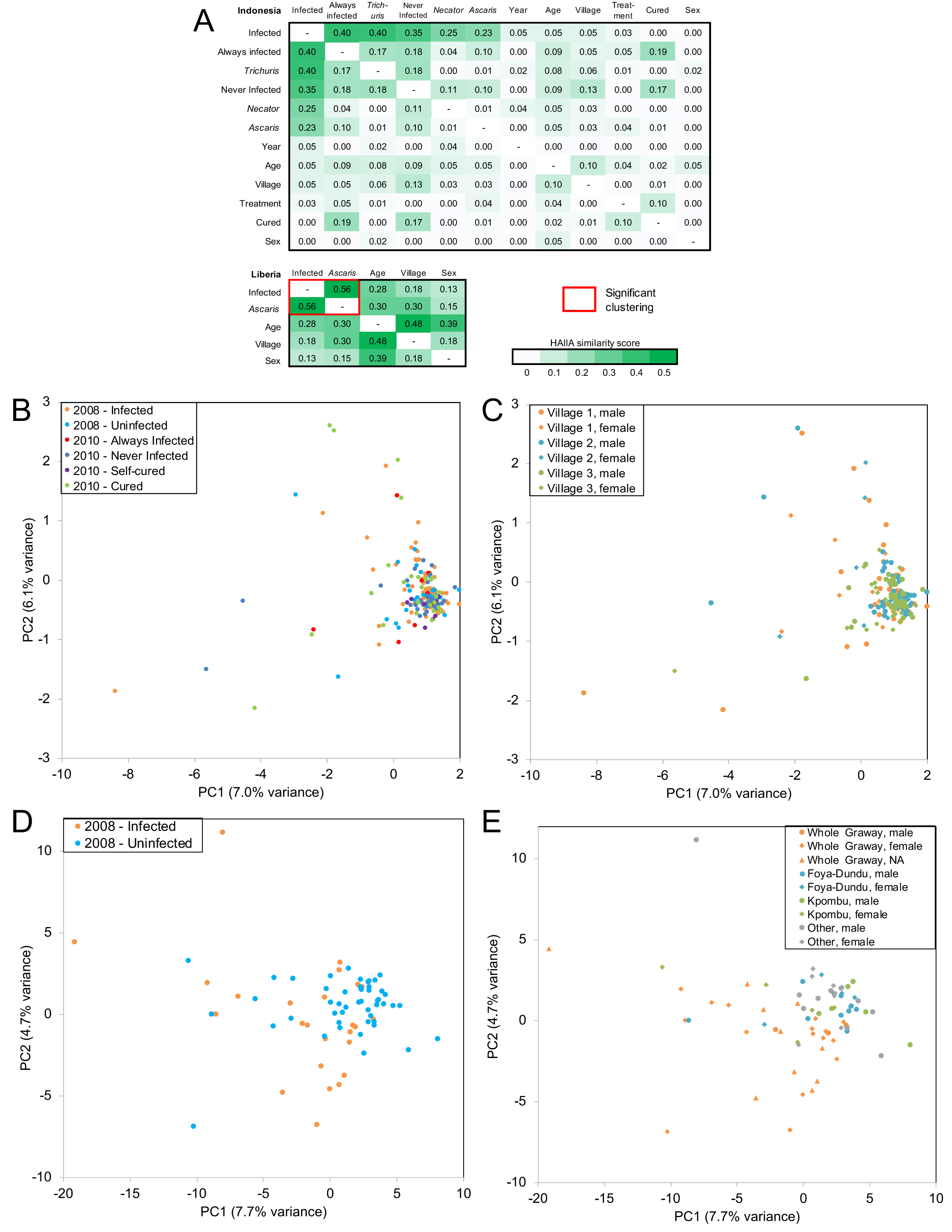

Supplement: Supplementary file 6 — Metadata analysis for Indonesia and Liberia datasets. (A) Hierarchical All-against-All (HAIIA) significance testing for metadata. (B–E) Principal component analysis (PCA) plots based on relative taxa abundance for all taxa identified in three or more samples, among heavy-infected or non-infected samples, are shown for Indonesia, with color coding according to (B) comparison cohort and (C) village and sex metadata, and for Liberia, with color coding according to (D) infected vs uninfected samples and (E) village and sex metadata. No significant differential clustering was identified, according to PERMANOVA. (TIFF 429 kb) [file 40168_2018_416_MOESM6_ESM.tif]

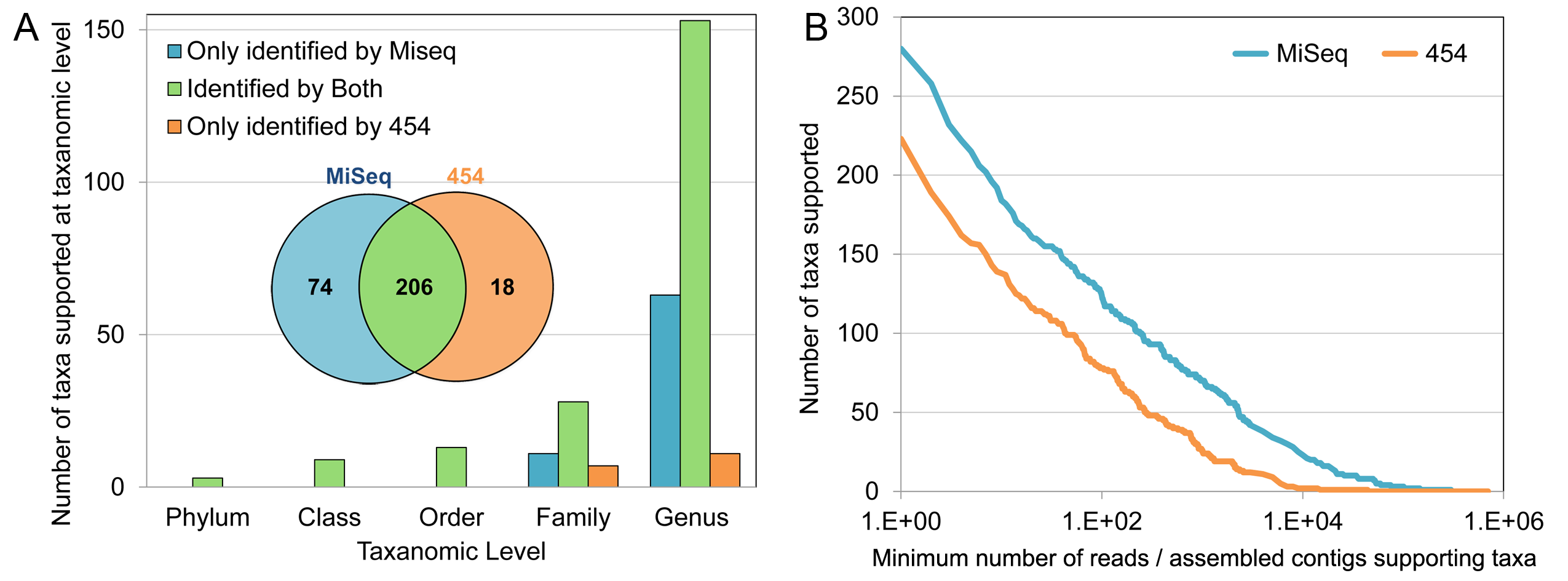

Supplement: Supplementary file 7 — Comparison of MiSeq and 454 sequencing platforms. (A) MiSeq identifies more unique bacterial taxa to the genus and family than 454. (B) Identified taxa are supported by more reads with MiSeq than with 454, improving statistical comparison power. (TIFF 334 kb) [file 40168_2018_416_MOESM7_ESM.tif]

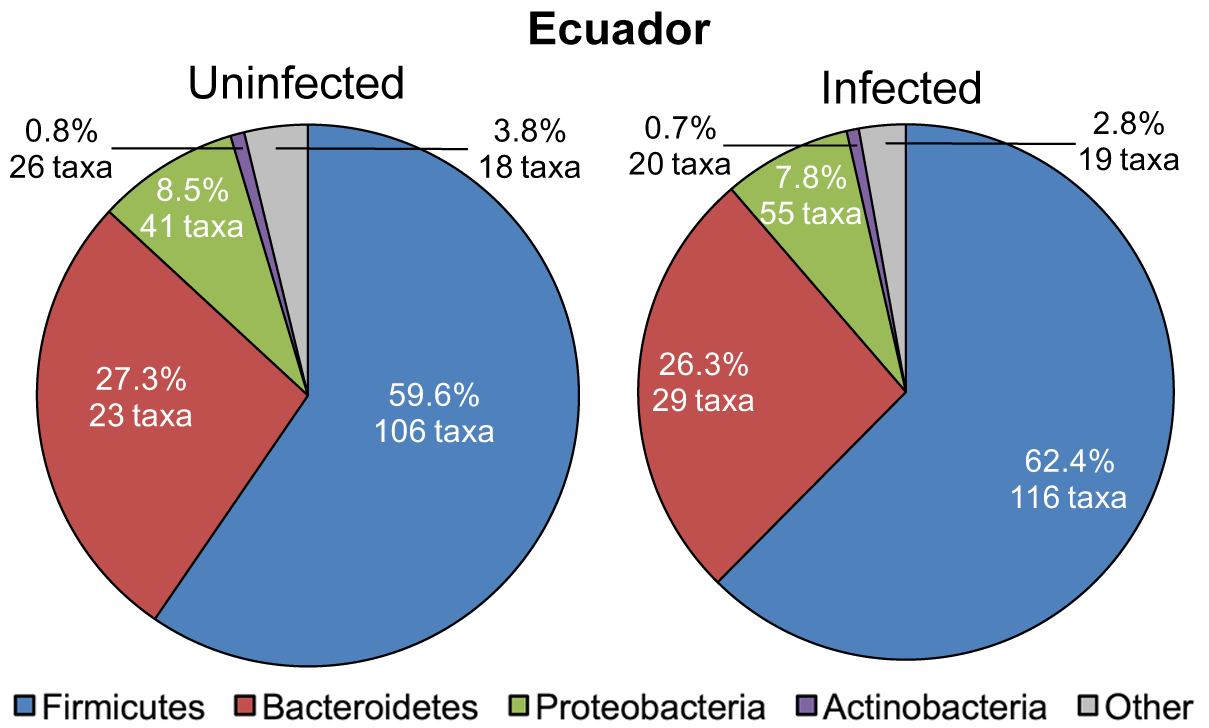

Supplement: Supplementary file 8 — Relative phylum abundance and taxa counts for the Ecuador sample set [27]. (TIFF 146 kb) [file 40168_2018_416_MOESM8_ESM.tif]
